# Coherent anti-Stokes Raman scattering enhancement of thymine adsorbed on graphene oxide

**DOI:** 10.1186/1556-276X-9-263

**Published:** 2014-05-27

**Authors:** Galyna Dovbeshko, Olena Fesenko, Andrej Dementjev, Renata Karpicz, Vladimir Fedorov, Oleg Yu Posudievsky

**Affiliations:** 1 Institute of Physics, National Academy of Sciences of Ukraine, 46 Nauki Ave, Kyiv 03680, Ukraine; 2 Institute of Physics, Center for Physical Sciences and Technology, A. Goštauto 11, Vilnius LT-01108, Lithuania; 3 Nikolaev Institute of Inorganic Chemistry, Siberian Branch of RAS, Akad. Lavrentiev Ave. 3, Novosibirsk 630090, Russia; 4 L.V. Pisarzhevsky Institute of Physical Chemistry, National Academy of Sciences of Ukraine, 31 Nauki Ave, 03028 Kyiv, Ukraine

**Keywords:** Graphene oxide (GO), Graphene nanoplatelets (GNPs), Multiwall carbon nanotubes (MWCNTs), Highly oriented pyrolytic graphite (HOPG), Thymine, Surface-enhanced Raman scattering (SERS), Coherent anti-Stokes Raman scattering (CARS), Surface-enhanced coherent anti-Stokes Raman scattering (SECARS), Graphene oxide-enhanced coherent anti-Stokes Raman scattering (GECARS)

## Abstract

Coherent anti-Stokes Raman scattering (CARS) of carbon nanostructures, namely, highly oriented pyrolytic graphite, graphene nanoplatelets, graphene oxide, and multiwall carbon nanotubes as well CARS spectra of thymine (Thy) molecules adsorbed on graphene oxide were studied. The spectra of the samples were compared with spontaneous Raman scattering (RS) spectra. The CARS spectra of Thy adsorbed on graphene oxide are characterized by shifts of the main bands in comparison with RS. The CARS spectra of the initial nanocarbons are definitely different: for all investigated materials, there is a redistribution of D- and G-mode intensities, significant shift of their frequencies (more than 20 cm^-1^), and appearance of new modes about 1,400 and 1,500 cm^-1^. The D band in CARS spectra is less changed than the G band; there is an absence of 2D-mode at 2,600 cm^-1^ for graphene and appearance of intensive modes of the second order between 2,400 and 3,000 cm^-1^. Multiphonon processes in graphene under many photon excitations seem to be responsible for the features of the CARS spectra. We found an enhancement of the CARS signal from thymine adsorbed on graphene oxide with maximum enhancement factor about 10^5^. The probable mechanism of CARS enhancement is discussed.

## Background

Enhancement of optical signals (Raman scattering, infrared absorption (IR), and luminescence) from molecules adsorbed on the surface of nanostructured metals was considered in many papers published recently. The nanostructured gold, platinum, silver, copper, and other metals were used for the achievement of the enhancement effect. The enhancement factor could achieve 10^6^ for Raman scattering and 10^3^ for IR absorption and luminescence [1,2]. Moreover, surface-enhanced Raman scattering (SERS) effect allowed registration of the signal from a single molecule adsorbed on the nanostructured surface [3]. The mechanism of this effect possesses dual electromagnetic (EM) and chemical (CM) nature and is the matter of debate in the literature [1-4].

Earlier, we have registered enhancement in Raman and IR spectra of different biomolecules adsorbed on carbon nanostructures: single-wall carbon nanotubes (SWCNTs) and graphene nanoflakes [5-7]. The maximum enhancement factor for Raman scattering of such nucleobases as thymine and adenine adsorbed on SWCNT was 10. It could be up to 80 on graphene oxide (GO) [8]. It is known from the literature that graphene could be used as enhancing support with enhancement factor from 17 to 69 [9-11].

The coherent anti-Stokes Raman scattering (CARS) technique is rather complex [12-14], and we found only a few papers devoted to its application for studying biomolecules [15-18]. The enhancement of CARS signal for molecules localized on nanostructured gold surface with an enhancement factor of approximately 10^5^ was published in [17]. It was also established that this method is attractive for visualization of macromolecules and cell components [19].

In the present paper, we used CARS to study different carbon nanostructured materials (highly oriented pyrolytic graphite (HOPG), multiwall carbon nanotubes (MWCNTs), graphene nanoplatelets (GNPs), and GO) as well as the surface-enhanced coherent anti-Stokes Raman scattering (SECARS) effect for thymine (Thy) adsorbed on GO.

## Methods

### Samples

Thy was purchased from Sigma-Aldrich (St. Louis, MO, USA) and used as received. The MWCNTs (Spetsmash, Kiev, Ukraine) have been synthesized by CVD method using Al_2_FeMo_0,21_ as a catalyst. The carbon content in the sample was 99.2% with soot as a residue; the catalyst was not found. The diameters of the MWCNTs varied from 2 to 40 nm; the surface area was 350 m^2^/g. The material has been certified by high-resolution transmission electron microscopy and Raman scattering [20].

GO was synthesized from graphite using mechanochemical approach to exfoliate microflakes accordingly [21]. During the synthesis, sulfuric acid was added to the mixture of the graphite microflakes (#043480, Alfa Aesar, Ward Hill, MA, USA) and KMnO_4_ as an oxidant and then it was mechanochemically treated using a planetary ball mill. The product of the mechanochemical treatment was washed on a glass filter by distilled water to remove the residues of the reagents soluble in water and undesirable products of the oxidation reaction, then by aqueous hydrochloric acid to remove manganese oxides insoluble in water, which were formed as a result of reduction of KMnO_4_, and finally with water to remove the residue of the acid. The product was placed in water where it quickly swelled and formed a stable dispersion of GO which was used thereafter. The prepared GO had C:H:O equal to 1.2:0.58:1.0 and an absorption maximum in UV-vis spectrum at 230 nm. It consisted of mono- and few-layered particles according to AFM and possessed photoluminescence with maximum of about 450 nm.

We used the GNPs produced by the Nikolaev Institute of Inorganic Chemistry, Siberian Branch of RAS (Novosibirsk, Russia). In accordance with the data of X-ray analysis and Raman spectroscopy, the GNPs predominantly consisted of 10 to 15 graphene layers with partial contribution of two- to three-layered nanoparticles. The lateral size of the GNPs was in the range from 5 to 9 μm [22]. The graphene monolayer on Cu foil was purchased from Aldrich, and HOPG was produced by State Scientific Research Institute of Structural Graphite Based Materials ‘NII Graphite’ (Moscow, Russian Federation).

The stock aqueous solution of Thy (1 mg/ml) was first prepared and then divided into two aliquots. One part of the solution was taken for further experiments. Another part of the stock solution was ultrasonically mixed (15 min), with a definite amount of the GO to obtain Thy/GO = 100:1 weight ratio. The samples for further studies were prepared by depositing a drop of Thy or Thy/GO solution on a glass substrate for CARS and on a metallic surface for the Raman experiments.

### Raman measurements

The Raman spectra of the monolayer graphene on Cu and HOPG were registered by inVia Raman microscope (Renishaw, Wotton-under-Edge, UK) using a laser with 633-nm wavelength and spot size of 1 μm. The Raman spectra of the MWCNTs, GO, and GNPs were also registered by inVia Raman microscope (Renishaw) using a diode laser with a wavelength of 785 nm. The SERS analysis of Thy/GO and Thy/MWCNT complexes was performed using the same laser. The band of Si at 520 cm^-1^ was used as the reference for wavenumber calibration. The WiRE 3.4 software (Renishaw) was used for Raman data acquisition and data analysis.

Carbon materials can be effectively characterized by Raman spectroscopy. The main feature of Raman spectra of graphite structure is the so-called ‘G band’ (1,600 cm^-1^) with *E*_1g_ band symmetry [23] in the Γ point of the Brillouin zone that correlates with the ordering of graphite crystal lattice. The second feature of graphite-like materials is the so-called ‘D band’ that characterizes the disorder of graphene layer lattice [24]. It refers to breathing vibrations of rings of graphene layer in the K point of the Brillouin zone. The second-order mode of this vibration (2D band) is registered at 2,600 to 2,700 cm^-1^, and it has an intensity which usually exceeds that of the second-order vibrations [25]. The last fact could be the evidence of carbon nanostructures consisting of similar structures that manifest a strong electron-phonon interaction and strong dispersion dependence of D-mode [24,25]. The characteristic feature of the Raman spectra of MWCNTs is that the halfwidth is equal to 50 cm^-1^ for the G-mode and above 60 cm^-1^ for the D-mode, and the D/G intensity ratio is greater than 1. The position of the G and D bands, appearance of breathing mode and its position, halfwidth, and relative intensity of all the bands could be used for the characterization of the nanotubes and their diameters.

The Raman spectrum of the graphene monolayer contains G and 2D bands analogous to graphite. The Raman spectrum of the GNPs and GO contains G, D, and 2D bands analogous to MWCNTs. The position of the 2D band maximum could be used as a characteristic to determine the number of layers in the graphene sheets [26].

### CARS measurements

**Figure 1:**
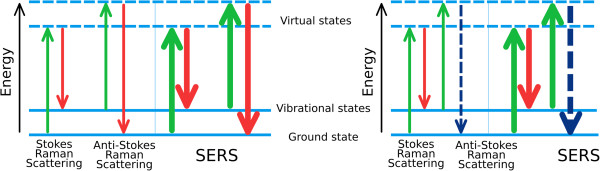
**Schematic band energy diagram showing transitions in different Raman processes.** In CARS, the pump (green arrow) and the Stokes (red arrow) beams drive the molecular vibrations. Through further interaction with the pump (another green arrow) beam, the blue-shifted photon (blue arrow) is emitted and detected.

CARS phenomenon is based on nonlinear interaction of two incoming optical fields on frequency *ω*_p_ (pump) and *ω*_S_ (Stokes) with material, which results in the generation of blueshifted anti-Stokes light with frequency *ω*_AS_ = *2ω*_p_ - *ω*_S_. Enhancement of the field on frequency *ω*_AS_ takes place when the frequency difference 2*ω*_p_ - *ω*_S_ coincides with the frequency of molecular vibrations of the studied material. Thus, tuning *ω*_p_ while keeping *ω*_S_ constant and detecting anti-Stokes light intensity, we could obtain CARS spectra containing information about the vibrational spectrum of the material. By spatial scanning the considered object at some fixed *ω*_AS_, we obtain a high-resolution image of the spatial distribution of the molecules possessing this particular vibrational band (Figure 1).

The experimental setup was described elsewhere [27]. Briefly, it is based on a home-made CARS microscope with compact laser source (EKSPLA Ltd., Vilnius, Lithuania). The laser consists of a picosecond (6 ps) frequency-doubled Nd:YVO_4_ pump laser with a pulse repetition frequency of 1 MHz and equipped with a travelling wave optical parametric generator (OPG) with a turning range from 690 to 2,300 nm. For CARS implementation, the OPG radiation was coupled with a fundamental laser radiation (1,064 nm) used as pump and Stokes excitation beams, respectively. Such mixing provides probing within the 700 to 4,500 cm^-1^ range of vibration frequencies. Both Stokes and pump beams were collinearly combined and directed to an inverted microscope (Olympus IX71, Center Valley, PA, USA). A spatial filter was used to improve the beam profile before directing into the microscope. The excitation light was focused on the sample with an oil immersion objective (Plan Apochromat, ×60, NA 1.42, Olympus). In the forward detection scheme, the CARS light was collected by another objective with NA 0.4. Long-pass and short-pass filters were used as blocking tools for spectral separation of the CARS signal. CARS radiation was detected using the avalanche photodiode (SPCM-AQRH-14, Perkin Elmer, Waltham, MA, USA) connected to a multifunctional board PCI 7833R (National Instruments Ltd. Dresden, Germany).

Measurements of the CARS spectra were performed in high-wavenumber region of Raman spectrum by tuning the OPG frequency (Table 1). In order to account for the spectral dependence of the OPG generation efficiency, the CARS signal intensity was normalized to the second power of the OPG radiation intensity. The spectral resolution of the CARS setup was approximately 8 cm^-1^. The spectra were recorded with a typical detection rate of 5 cm^-1^/s.

**Table 1:** Operating CARS frequency

**CARS registration range (cm**^**-1**^**)**	**Stokes (nm)**	**Pump (nm)**	**Anti-Stokes (or CARS) (nm)**
1,200 to 1,700	1,064	940 to 900	850 to 780
2,500 to 3,500	1,064	840 to 775	690 to 610

A Piezo scanning system (Physik Instrumente GmbH & Co., Karlsruhe, Germany) was used for scanning the samples. Images of 250 × 250 pixels were obtained with 2-ms pixel dwell time. Excitation pulse energies from 1 to 10 nJ of the samples for both pump and Stokes beams were used. Sample scanning, data processing, and laser wavelength tuning were controlled with a computer. The excitation light was focused on the sample with an oil immersion objective (Plan Apochromat, ×60, NA 1.42, Olympus). This numerical aperture of the focusing objective provides tight focusing of NIR exciting light with effective lateral point spread function of about 0.4 μm. The corresponding axial point spread function is about 1.0 μm. Thus, the CARS images in this paper have resolutions of approximately 0.5 μm in the *X* and *Y* directions, and approximately 1.0 μm in the *Z* direction.

## Results and discussion

### Raman and CARS spectra of the carbon materials

**Figure 2:**
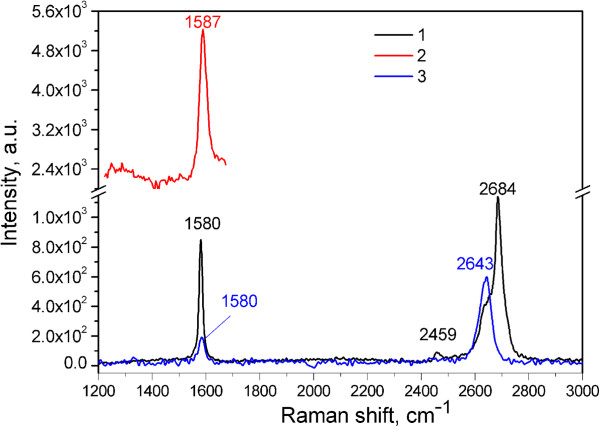
**Raman spectra of HOPG and monolayer graphene and CARS spectrum of HOPG.** Raman spectra of HOPG (1) and monolayer graphene on Cu (3) at *λ*_ex_ = 633 nm. CARS spectrum of HOPG (2).

The CARS and Raman spectra of the different carbon materials such as HOPG and monolayer graphene on Cu are presented in Figure 2 for comparison. The CARS spectra of the graphene monolayer on Cu foil could not be registered due to technical reasons; it was wrapped and burned. It is seen that the position of the G-mode (1,580 cm^-1^) for HOPG and monolayer graphene is approximately the same with that in the Raman spectra. However, a definite high-frequency shift of 7 cm^-1^ is observed for this mode in the CARS spectrum of HOPG. The position of 2D-mode maximum in the Raman spectrum of HOPG is blueshifted by 41 cm^-1^ relatively to that of the monolayer graphene, the form of the band being asymmetric with a shoulder at approximately 2,640 cm^-1^, a characteristic position of the band for the monolayer graphene.

**Figure 3:**
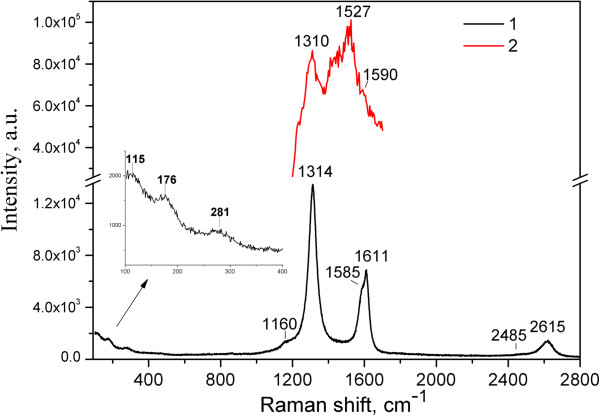
**Raman (1) at λ**_**ex**_** = 785 nm and CARS (2) spectra of MWCNTs.**

The CARS and Raman spectra of MWCNTs are presented in Figure 3. The band in the Raman spectrum of MWCNTs about 1,600 cm^-1^ is asymmetric, consisting of G-mode at 1,585 cm^-1^ and D′-mode at 1,611 cm^-1^. The G-mode in the CARS spectrum of MWCNTs is seen as a weak shoulder only (Figure 3) as compared with the strong new band at 1,527 cm^-1^ (denoted here as G_CARS_) and the shoulder at 1,416 cm^-1^. In contrary to the Raman and CARS spectra of HOPG, the spectrum of MWCNTs contains D-mode which is indicative of the presence of defects. The Raman spectrum also contains several low-frequency modes (inset in Figure 3) whose positions could be used to determine the internal and external diameters of the nanotubes.

**Figure 4:**
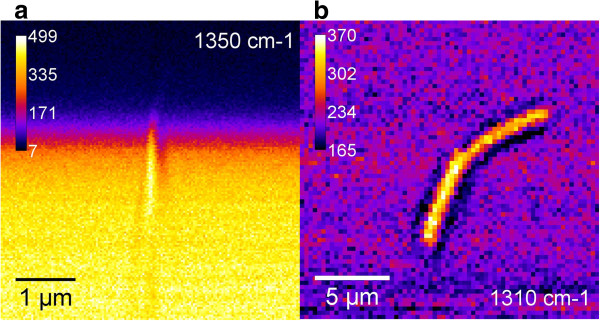
**CARS images at 1,350 cm**^**-1 **^**(a) and 1,310 (b) cm**^**-1 **^**of MWCNTs.**

The images of the MWCNTs obtained using D-mode at 1,310 cm^-1^ are shown in Figure 4. Since CARS is a four-wave mixing (FWM) process, there are two contributions to the measured anti-Stokes signal: vibrational and electronic. The CARS spectrum of the MWCNTs has no distinct vibrational bands (Figure 3). That means that the contrast of the image has a predominantly electronic nature in accord with the earlier observations of the SWCNTs by FWM microscopy [28]. Moreover, in our case, the MWCNTs are located on the glass surface, and the scanning beam probes captured not only the MWCNTs but also the glass, so the contribution from the glass reduces the image contrast (Figure 4). Nevertheless, the lateral image recorded at the fixed value of *z* coordinate possesses a rather good contrast which allowed us to identify reliably the size of MWCNTs (Figure 4a,b). It appeared to be equal approximately to 15 μm in length and approximately 250 nm in width. The image of the MWCNTs has the same intensity throughout the length which indicates a uniform distribution of defects.

**Figure 5:**
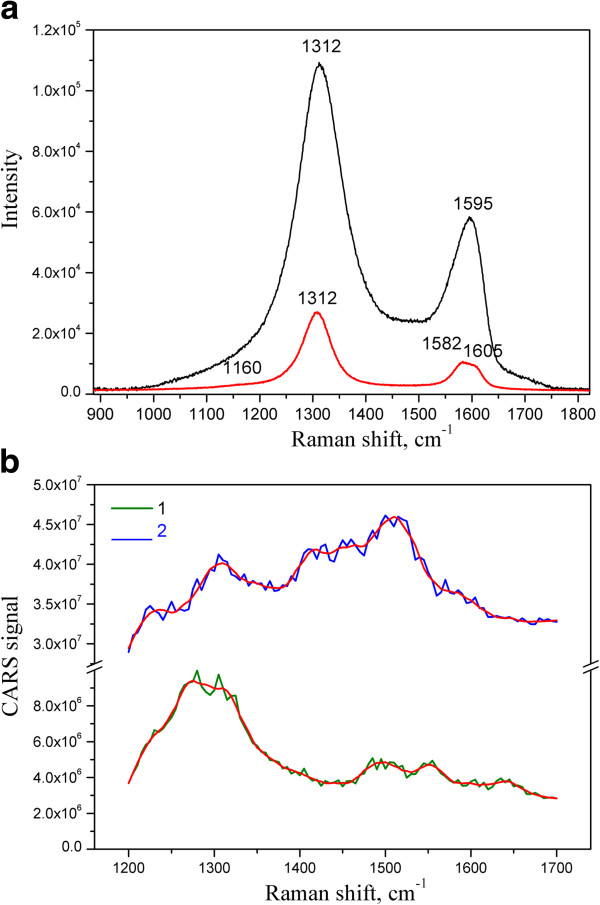
**Raman at *λ***_**ex**_** = 785 nm (a) and CARS (b) spectra of GNPs (1) and GO (2).**

The CARS and Raman spectra of the GNPs and GO are presented in Figure 5. It could be seen that the spectra are definitely different from each other for both carbon materials. For instance, the G-mode in the Raman spectrum of the GNPs is at 1,582 cm^-1^, whereas in the CARS spectrum, it is shifted to 1,555 cm^-1^. It is obviously strong and located at 1,595 cm^-1^ in the Raman spectrum of the GO, whereas it is about 1,584 cm^-1^ in the CARS spectrum in a form of a weak shoulder on the background of the strong band at 1,516 cm^-1^. Analogously to the CARS spectrum of MWCNTs (Figure 3), the highly intensive G_CARS_-mode at 1,516 cm^-1^ in the CARS spectrum of GO is observable. New arising bands at 1,419 and 1,516 cm^-1^ in GO and at 1,500 and 1,555 cm^-1^ in GNPs could be assigned to the vibrations from the edge atoms, and also according to [14], the first principal calculation showed new emerging bands at 1,450 and 1,530 cm^-1^.

The position of D-mode in CARS and Raman spectra is approximately the same. Besides, it is worthwhile to mark the widening of the D-mode in the case of the CARS spectra of GNPs and the redistribution between *I*_D_ and *I*_G_ in the CARS spectra relatively to the Raman analogues.

**Figure 6:**
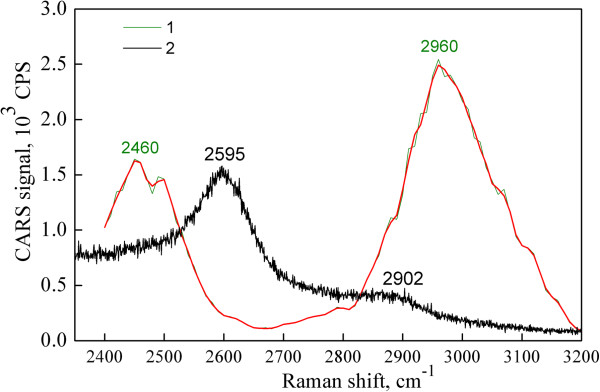
**CARS (1) and Raman at *λ***_**ex**_** = 785 nm (2) spectra of GNPs.**

Another feature of the interrelation between Raman and CARS spectra is observed in the 2,400 to 3,200 cm^-1^ range. The corresponding spectra of the GNPs are presented in Figure 6. It is seen that the Raman spectrum of the GNPs has a usual form, as represented by the strong 2D-mode at 2,595 cm^-1^. At the same time, this mode is absent in the CARS spectrum, while there appeared another two strong band frequencies which are 2,460 and 2,960 cm^-1^ (Figure 6). It could be supposed that the first is a combination of D-mode with a mode at approximately 1,150 cm^-1^ (D_1_) which corresponds to a phonon belonging to a point other than K and Γ of the Brillouin zone [29], and the second is probably a double resonance of the 1,516 cm^-1^ band. The disappearance of the 2D-mode is supposed to be connected with specificity of the CARS technique and the absence of the conditions for double electron-phonon resonance. Simultaneously, in the region of the second tones, we registered more bands than the usual, so multiphonon processes [30,31] could occur more efficiently.

The modes near 2,460 cm^-1^ as well as those in the region of 2,400 to 3,200 cm^-1^ are assigned to overtones [26]. Nemanich and Solin [24] have registered a band at 3,250 cm^-1^ and a weaker band at 2,450 cm^-1^ in the Raman spectra of graphite. The last band was named as D″ by Vidano and Fishbach [25,32]. Later, Nemanich and Solin, using polarization measurement, assigned the peaks in the 2,300- to 3,250-cm^-1^ region to overtones in graphite [24], and the 2,950-cm^-1^ band to D + D′ (D′-mode at 1,620 cm^-1^ is due to disorder) rather than to D + G. Vidano and Fishbach [25] confirmed that the 3,250-cm^-1^ band is the D′ overtone, analogous to the band at 2,700 cm^-1^ which is the D overtone named G′. Interestingly, those bands do not shift with excitation energy, and the energy dependence of the 2,950-cm^-1^ band is consistent with D + D′ or D + G.

**Figure 7:**
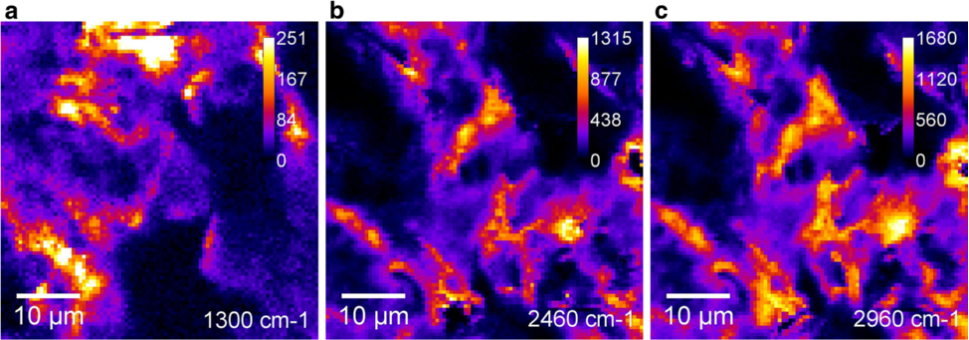
**CARS images of GNPs using the bands at 1,300 cm**^**-1 **^**(a)**, **2,460 cm**^**-1 **^**(b), and 2,960 (c) cm**^**-1**^**.**

The CARS images of the GNPs obtained using the different bands are presented in Figure 7. The distribution of the intensity of the CARS bands could be obviously seen: the intensities of the bands at 2,460 and 2,960 cm^-1^ are similar, where the intensity of the signal at 2,960 cm^-1^ is higher, so the image obtained using this band is brighter. Both the images and the spectrum of the GNPs have essentially vibrational origin. In accordance to [10] and Figure 7 (more bright imaging at the end of particles), we could suppose about the increase of the local electromagnetic field at the edges of the different graphene particles.

The modes found by using Raman and CARS spectroscopy in different carbon materials are summarized in Tables 2 and 3. Based on the presented data, it could be concluded that the position of the D-mode of the studied materials is close for Raman and CARS spectra; this is in contrary to that of the G-mode, which, in the CARS spectra, is significantly decreased on the background of the new intensive mode (G_CARS_), depending on the type of the carbon material.

**Table 2:** CARS bands of the different carbon materials

**Assignment**	**GNP (cm**^**-1**^**)**	**GO (cm**^**-1**^**)**	**MWCNT (cm**^**-1**^**)**	**HOPG (cm**^**-1**^**)**
D	1,300	1,306	1,310	Not detected
New band	Not detected	1,419	1,421	Not detected
New band	1,500	1,516	1,527	Not detected
G	1,555	1,584	1,590	1,587
D′	Not detected	Not detected	Not measured	Not measured
2D (G′)	Not detected	Not measured	Not measured	Not measured
D + D_1_	2,460	Not measured	Not measured	Not measured
2G_CARS_	2,960	Not measured	Not measured	Not measured

**Table 3:** Raman bands of the different carbon materials

**Assignment**	**GNP (cm**^**-1**^**)**	**GO (cm**^**-1**^**)**	**MWCNT (cm**^**-1**^**)**	**HOPG (cm**^**-1**^**)**
D-mode	1,307	1,312	1,314	Not detected
G-mode	1,582	1,595	1,589	1,580
D′	1,605	Not detected	1,611	Not detected
G′-mode (2D)	2,595	2,616	2,615	2,684
D + D′ (or D + G)	2,902	Not detected	Not detected	Not detected

### Raman and CARS spectra of the Thy/GO complex

**Figure 8:**
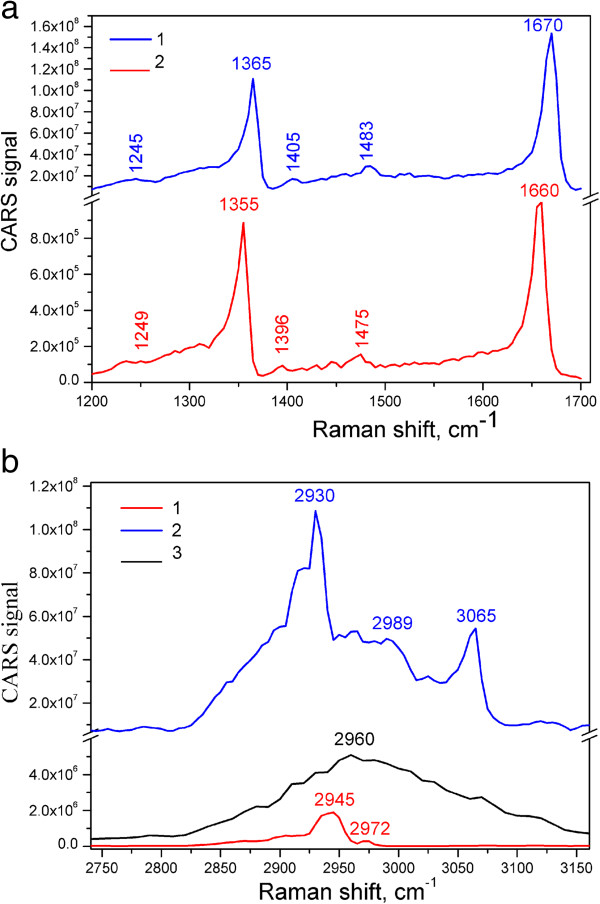
**CARS spectra of Thy**/**GO (1)**, **Thy (2) and GNPs (3).** CARS spectra of Thy/GO (1) and Thy (2) in 1,200 to 1,700 cm^-1^**(a)** and CARS spectra of Thy/GO (1), Thy (2), and GNPs (3) in 2,400 to 3,200 cm^-1^**(b)** ranges.

The CARS spectra of Thy and the Thy/GO complex are shown in Figure 8. It is seen that the bands of Thy were shifted from 1,355 and 1,660 cm^-1^ to 1,365 and 1,670 cm^-1^ in Thy/GO complex, correspondingly. It could be suggested that these high-frequency shifts are due to the interaction of Thy with carboxyl and hydroxyl groups of GO [33]. The redistribution of the intensity of the bands and a new mode at 3,065 cm^-1^ are characteristic of Thy/GO complex. Taking into account the presence of the wide band at 2,960 cm^-1^ in the CARS spectrum of GNPs (Figure 6), it could be assumed that the widening of the CARS spectrum of Thy/GO complex is an evidence of the electron-phonon and phonon-phonon resonances [34]. The intensity of the CARS signals of the Thy/GO complex exceeds the CARS signals of Thy at more than 10^4^ times.

**Figure 9:**
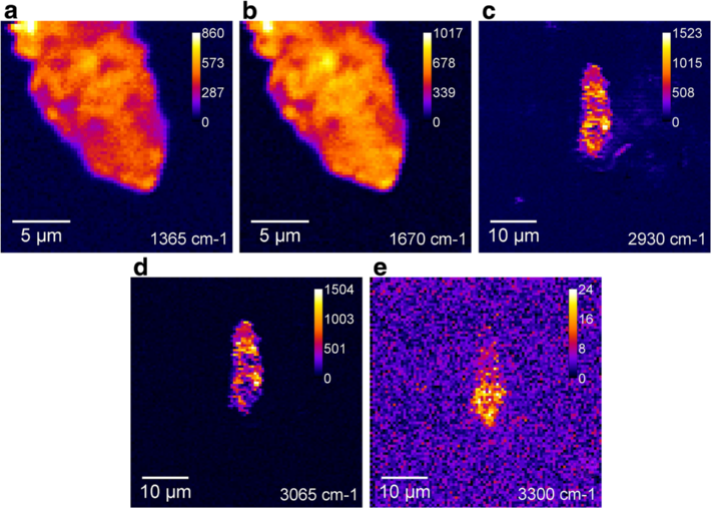
CARS (a,b,c,d,e) images of the Thy/GO complex.

The study of the Thy/GO complex by CARS spectroscopy was carried out in two spectral ranges. Both fingerprint and high-frequency ranges revealed strong bands belonging to Thy. The vibrational contribution to the spectra is dominating, so obtaining high-quality vibrational images of the complex is a possibility. The CARS images of Thy/GO recorded at several wavenumbers are shown in Figure 9. The bands at 1,365 and 1,670 cm^-1^ and at 2,930, 3,065, and 3,300 cm^-1^ are used to obtain the images of two different fragments of the sample. Scans at 2,930, 3,065, and 3,300 cm^-1^ were done in 50 × 50-μm area and show the typical fragment entirely. All images have a very high contrast with respect to the image at 3,300 cm^-1^, where the background at non-resonance wavenumber is shown. It should be mentioned on the basis of comparison (Figure 9a,c) that the intensity of the CARS band at 2,930 cm^-1^ of Thy/GO is higher than that at 1,365 cm^-1^ (one of the most intensive bands). This fact supports our assumption regarding the interaction between Thy and GO modes.

So, from the CARS images, it is seen that the Thy/GO complex adsorbed on the glass surface is not as a solid film but rather as flat flakes with lateral size from 1 to 15 μm. It is important to note that the most intensive CARS bands of GNPs and Thy/GO are, respectively, at 2,960 and at 2,930 cm^-1^. So, it could be supposed that the enhancement of the CARS bands of the Thy/GO complex in the 2,930- to 3,100 cm^-1^-range is connected with the chemical interaction between Thy and GO.

**Figure 10:**
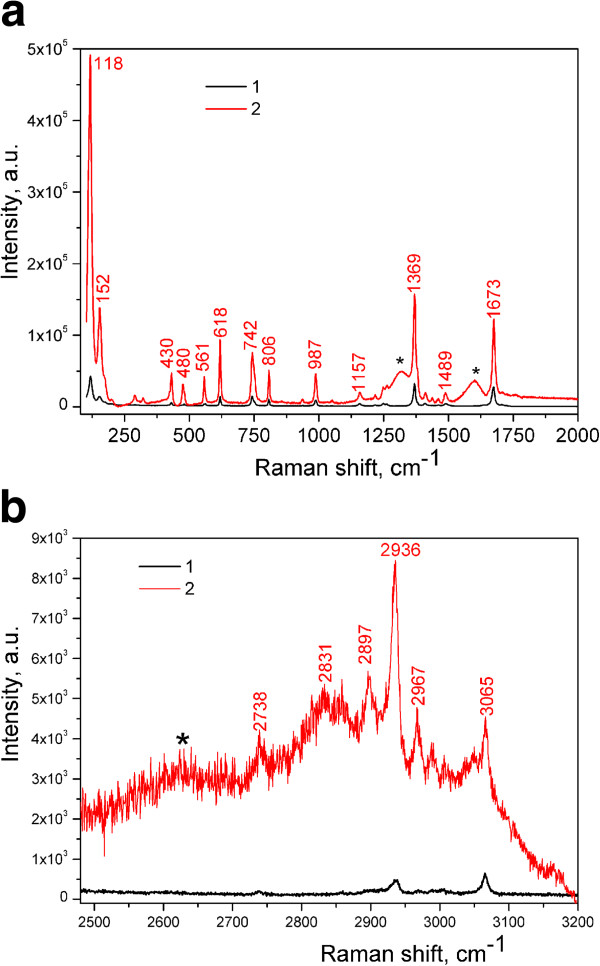
**Raman spectra of Thy (1) and Thy/GO (2) at *λ***_**ex**_** = 785 nm. (a)** In 1,200 to 1,700 cm^**-**1^ range. **(b)** In 2,400 to 3,200 cm^-1^ range. The modes of GO are labeled by asterisks (*).

The Raman spectra of Thy and the Thy/GO complex are shown in Figure 10. In the spectra of Thy/GO, the characteristic bands of GO (D-, G-, and 2D-modes) are clearly seen. Also, in the 2,750- to 3,200-cm^-1^ range, the enhancement and widening of the characteristic bands of Thy are observed. Importantly, these bands are the features of the CARS spectra as well (Figure 8).

The assignment of Raman and CARS spectral bands for Thy and Thy/GO complex is presented in Table 3. As a whole, the position of the bands in the Raman and CARS spectra is often close. In the CARS spectrum of the Thy/GO complex, there are NH and CH stretching modes in the 3,000- to 3,300-cm^-1^ range, and the C_6_H stretching modes of medium intensity are at 3,065 cm^-1^. It is interesting that in the CARS spectra of the Thy/GO complex (Table 4), there is only one band at 1,670 cm^-1^, whereas in the corresponding spectra of Thy, there are two bands at 1,655 and 1,660 cm^-1^, attributed to C_4_O and C_2_O stretching modes, respectively. A similar effect was observed in the case of SERS of Thy on gold in comparison with RS of those [35]; however, its nature could have another origin. It depends on the peculiarities of the CARS method and orientation of Thy in relation to graphene oxide surface.

**Table 4:** **Assignment of the spectral bands (cm**^-**1**^**) observed for Thy and Thy**/**GO complex**

**RAMAN (*λ***_**ex**_ **= 785 nm) spectra**		**CARS spectra**		**Assignment in Thy**[36-38]**and GO**[33]
**Thy**	**Thy/GO**	**Thy**	**Thy/GO**	
-	-	-	3,300	ν (OH) in GO
-	-	-	3,167	ν (NH); ν (OH) GO
3,065	3,065	-	3,065	ν (C_6_H)
3,006	3,005	-	2,991	ν (CH_3_)
2,968	2,967	-	2,963	ν (CH_3_)
2,989	2,989	2,972	2,989	ν (CH_3_)
2,936	2,936	2,945	2,930	ν (CH_3_)
2,892	2,897	2,870	-	ν (CH_3_)
-	2,831	-	2,848	CH, GO
2,739	2,738	2,749	2,753	CH
-	2,626	-	-	2D-mode in GO
1,673	1,675	1,660	1,670	ν (C_2_ = O)
-	-	1,655	-	ν (C_4_ = O)
-	1,603	-	-	G-mode in GO
-	-	1,520	1,525	ν ring
1,489	1,490	1,482	1,483	δN_1_H
1,460	1,459	1,461	1,458	δ_as_ (CH_3_)
1,437	-	-	1,436	CH_3_
1,411	1,410	1,406	1,405	νC2N3, δN_1_H
1,368	1,368	1,355	1,365	δ_s_ (CH_3_), δ (N_3_H)
-	1,319	-	-	D-mode in GO
1,247	1,248	1,247	1,249	ν (ring)

ν, stretching; δ, deformation*.* All bands are assigned to Thy; the bands assigned to graphene oxide are noted.

To determine the enhancement factor of the CARS signal for the Thy/GO complex relative to Thy, the filling factor and the conditions of the CARS experiment should be evaluated. In CARS experiments, the radiation comes from the space volume of approximately 1 μm^3^. Such volume can contain approximately 10^9^ molecules of Thy (without graphene). When GO is added to Thy, in accord with our estimation, the number of Thy molecules within the mentioned volume is approximately 10^8^. Then, taking into account these assumptions and the difference between the intensity of the CARS signal for the Thy/GO complex and Thy from Figure 8 (approximately 10^4^), we could obtain that the CARS enhancement factor is equal to approximately 10^5^. The enhancement obviously arises from those molecules of Thy which are in close proximity to the surface of GO. The number of such Thy molecules is really lower than the whole number of the molecules in the volume. So, the obtained estimation of the enhancement factor should be considered as the lower limit. It could also be mentioned that the value of the enhancement factor is not the same for the whole range from 1,200 to 3,300 cm^-1^. It is the maximum for the NH and CH stretching modes which usually appear in 3,000- to 3,200-cm^-1^ range (Figure 8b).

The enhancement effect of the CARS spectrum of the Thy/GO complex seems to be similar to that of SECARS (Figure 8), and it could be named as graphene oxide-enhanced CARS (GECARS), analogous to the graphene-enhanced Raman scattering (GERS) technique, in which graphene can be used as a substrate for SERS of adsorbed molecules [9,11,39].

SERS enhancement is typically explained by CM [40] and EM [1,41-43] mechanisms. CM is based on charge transfer between the probed molecule and the substrate. On the other hand, the origin of EM mechanism is connected with great increase of the local electric field caused by plasmon resonance in nanosized metals, such as Ag and Au [41]. These two mechanisms always contribute simultaneously to the overall enhancement, and it is usually thought that EM provides the main enhancement. For graphene-type materials, due to the fact that surface plasmon in graphene is in terahertz range rather than within the range of visible light [44], GERS, in most cases, does not support the EM mechanism, and it is more appropriate to consider the CM. However, in the case of the GO, the oxygen-containing groups could create strong local electric field [45] under laser excitation, so large polarizability of graphene domains induces additional local electric field and increases the cross-section of RS of the adsorbed molecules. Additional enhancement could be explained by resonant excitation for one or two photons in the case of CARS of nanocarbons (Table 1) also. Indeed, our optical study in the near-visible range confirms the appearance of local density states of MWCNTs and GNPs in the region of 500 to 900 nm. So, resonant excitation could be the other reason of giant enhancement in CARS. All this mechanisms need further study and analysis.

## Conclusions

Therefore, it was shown that the CARS spectra of carbon nanostructures (GNPs, GO, and MWCNTs) are definitely different from the corresponding spontaneous Raman spectra. At the same time, the CARS and Raman spectra of Thy are rather close and could be used for analytical purposes. The GECARS effect was shown for the Thy/GO complex with minor shifts of Thy bands. The enhancement factor of the GECARS signal for the Thy/GO complex is greater than approximately 10^5^. In our view, the enhancement effect could have several reasons: (a) the so-called chemical mechanism, which involves charge transfer between the molecule and the carbon nanostructure, as well as the increase of the dipole moment in the molecule; (b) the resonant interaction of exciting light with electronic states of the carbon nanostructures; and (c) the increase the local electromagnetic field at the edges of the GO nanosheets.

## Abbreviations

CARS: coherent anti-Stokes Raman scattering; CM: chemical mechanism; EM: electromagnetic mechanism; GECARS: graphene oxide-enhanced coherent anti-Stokes Raman scattering; GNP: graphene nanoplatelet; GO: graphene oxide; HOPG: highly oriented pyrolytic graphite; IR: infrared spectroscopy; MWCNT: multiwalled carbon nanotube; SERS: surface-enhanced Raman scattering; SECARS: surface-enhanced coherent anti-Stokes Raman scattering; SWCNT: single-walled carbon nanotubes.

## Competing interests

The authors declare that they have no competing interests.

## Authors' contributions

GD has given final approval of the version to be published. OF has made substantial contributions to the conception and design of the study and acquisition, analysis, and interpretation of data. AD has been involved in drafting the manuscript and in measuring the CARS spectra. RK has been involved in drafting the manuscript. VF carried out the synthesis of the graphene nanoplatelets. OP carried out the synthesis of graphene oxide and participated in drafting the manuscript. All authors read and approved the final manuscript.

## Authors' information

GD has a scientific degree of Doctor of Sciences in Solid State Physics and Biophysics and received degree of professor in 2012. She is a Head of Physics of the Biological Systems Department of Institute of Physics of National Academy of Sciences of Ukraine. Her scientific areas of interest are Biophysics, nucleic acids, Solid State Physics, surface solids, plasmonics, experimental physics (FTIR, SEIRA, SERS, UV, Raman, NMR spectroscopy, Langmuir-Blodgett technique, AFM microscopy, and Computational Chemistry). She was involved in the study of biological molecule interaction with low doses of ionizing and microwave irradiation, ligands, anti-cancer drugs, metal and carbon nanostructures. She has more than 250 publications in international scientific journals. OF received her degree of Senior Researcher in 2009 and her Ph.D. at Institute of Physics of National Academy of Sciences of Ukraine in 2007 with a thesis about effects and mechanisms of enhancement of optical transition of bio-organical molecules near metal surface. Now, she is the Head of the Innovations and Technology Transfer Department of the Institute of Physics of National Academy of Sciences of Ukraine. Her present study mainly focuses on the spectroscopical manifestation of SEIRA, SERS, enhanced luminescence effect of biological molecules, and development of metal enhancing supports for application. In 2009, her work ‘The enhancement of the optical processes on the metallic surface and its application for the detection of small quantity of molecules and revealing the structure of tumors macromolecules’ was awarded with the Prize for Young Scientists from the President of Ukraine. She is currently managing FP7 Nanotwinning Project within the framework of which inVia Raman microscope (Renishaw) was purchased and is actively used in the experiments described in this article. OP is a Senior Research scientist in the Free Radicals Department of L.V. Pisarzhevsky Institute of Physical Chemistry of the National Academy of Science of Ukraine. He received his Ph.D. from L.V. Pisarzhevsky Institute of Physical Chemistry of the National Academy of Science of Ukraine in 1985. His research interests include preparation and physical chemistry of new functional materials, conducting polymers, graphene oxide, graphene and graphene-like nanomaterials, hybrid nanocomposites, sensors, lithium batteries, and light-emitting diodes. He is the author of more than 100 scientific publications. Also, he is a scientific referee of European FP6 and FP7, German-Israeli Foundation for Scientific Research and Development (GIF), numerous scientific journals published by Elsevier, Wiley, the Royal Society of Chemistry, and American Chemical Society. AD is a Ph.D. degree holder and a Senior Research Scientist in the Molecular Compounds Physics Department of State Research Institute Center for Physical Sciences and Technology. His main research interests include nonlinear optical microscopy, chemical imaging by means of coherent anti-Stokes Raman microscopy, application of coherent Raman microscopy to bio-objects, and optical nonlinearity of nanostructured organic polymers. He is a member of the management committee in COST Action ‘Chemical Imaging by Coherent Raman Microscopy – microCoR’ from Lithuania. RK works as a Senior Researcher in the Molecular Compound Physics Department at the Center for Physical Sciences and Technology. She defended her Ph.D. thesis in 2001 at the Institute of Physics, Vilnius. Her main research interests are spectroscopic characterization of organic materials, ultrafast excitation relaxation processes in organic molecular compounds, molecular isomerization, tautomerization, charge transfer processes, and charge carrier generation in organic semiconductors. She is the author of more than 25 scientific papers.

VF received the following scientific degrees: Ph.D. in 1966, Doctor of Chemical Science in 1990 and the title of Full Professor in 1991. He was awarded with the title of Honored Science Worker of Russian Federation (2004). Currently, he works as the Chief Scientist at Nikolaev Institute of Inorganic Chemistry, Siberian Branch of Russian Academy of Sciences. He is a lecturer at the Natural Science Department of the Novosibirsk State University. He actively collaborates with universities in many countries. His research interests lie in the fields of solid state chemistry, synthesis and materials design, and crystal and electronic structures of low-dimensional inorganic materials with unusual electronic properties. He has more than 400 publications, including original articles, reviews, patents, and three books.
